# The Relationship between Annual Airborne Pollen Levels and Occurrence of All Cancers, and Lung, Stomach, Colorectal, Pancreatic and Breast Cancers: A Retrospective Study from the National Registry Database of Cancer Incidence in Japan, 1975–2015

**DOI:** 10.3390/ijerph17113950

**Published:** 2020-06-02

**Authors:** Akira Awaya, Yoshiyuki Kuroiwa

**Affiliations:** 1 Dermatology & Epidemiology Research Institute (DERI), 4978 Totsuka-cho, Totsuka-ku, Yokohama, Kanagawa 244–0003, Japan; 2 Department of Genome System Science, Yokohama City University, Seto 22–2, Kanazawa-ku, Yokohama, Kanagawa 236–0027, Japan; 3 Department of Neurology and Stroke Center, University Hospital Mizonokuchi, Teikyo University School of Medicine, 5–1-1, Futago, Takatsu-ku, Kawasaki, Kanagawa 213–8507, Japan; ykuroiwa@med.teikyo-u.ac.jp; 4 Department of Medical Office, Ministry of Finance, Japanese Government, 3–1-1, Kasumigaseki, Chiyoda-ku, Tokyo 100–8940, Japan; 5 Department of Neurology, Yokohama City University Graduate School of Medical Sciences, 3–9, Fukuura, Kanazawa-ku, Yokohama 236–0004, Japan

**Keywords:** cancer incidence, airborne pollen exposure, lung cancer, pancreatic cancer, breast cancer, pollen diseases, Kawasaki disease, specific intractable diseases

## Abstract

Suppression of risk factors including smoking, overdrinking and infections by human papilloma and hepatitis B and C viruses has been recommended for cancer prevention; however, identification of other environmental risk factors has not been enough. Besides the 2003 report that Kawasaki disease may be triggered by pollen exposure, 40 Japanese specific intractable diseases have recently been reported as “pollen diseases”, also potentially triggered by pollen exposure. Various human organs are affected by pollen exposure, leading to systemic vasculitis; autoimmune connective tissue diseases, inflammatory bowel diseases and intractable neuromuscular and bone diseases, suggesting the common effects of pollen exposure on fundamental functions of vital metabolism. In this context, cancer and malignant tumors may be another group of intractable diseases triggered by epigenetic pollen exposure. Thus, this study compared the number of newly registered patients with 24 types of cancer and airborne pollen levels measured from 1975 to 2015. We searched for statistical correlations with Bonferroni correction between the annual number of newly registered patients for all cancers or for each of lung, stomach, colorectal, pancreatic and breast cancers in the patient-registry year “x”, and annual airborne pollen levels measured in the same year as “x”, or 1–7 years prior to the year “x”. The number of newly registered patients for lung, and pancreatic cancers in the patient-registry year “x” was highly correlated with airborne pollen levels measured 2 years prior to “x”. That for breast cancer was correlated with pollen levels measured 2 and 5 years prior to “x”. To our knowledge, this is the first rapid communication of the association between pollen levels and cancer incidence.

## 1. Introduction

Worldwide efforts seek to prolong human life and prevent deaths due to various cancers by developing anticancer agents and therapeutic methods. However, the reason for the increase in the number of cancer patients has been poorly understood so far. The cancer incidence has been registered in Japan since 1958 [1]. Data on these registered patients (RPs) reported by the National Cancer Center (NCC) has indicated an increase in cancer incidence since 1980, particularly after 1985 [1,2]. In spite of long-standing national education [3,4] on precautions for cancer prevention against excessive drinking, smoking and human papilloma and hepatitis B and C viral infections, the number of cancer patients has been soaring [1,2]. Thus, the authors have hypothesized that other causes besides these known risk factors for cancer, such as lifestyle behaviors, viral infections and gene mutations may trigger cancer. Sasazuki et al. [4] re-evaluated evidence-based recommendations for cancer prevention in Japan, including awareness of smoking, drinking, food, body weight, physical activity, infections and others; however, there appears to be limited consideration of other environmental and lifestyle factors. In particular, the effects of pollen cells, a product related to fertilization in the plant kingdom, on carcinogenesis in the animal kingdom, particularly humans, has received little consideration.

We previously conducted an epidemiological study [5,6,7,8,9] on Kawasaki disease (KD), a systemic vasculitis, to clarify the relationship between weekly, monthly, and annual occurrence of KD and weekly, monthly, and annual levels of airborne pollen (AP) released by Japanese cedar. We first noted that triphasic outbreaks of KD in all Japan, Tokyo and Kanagawa over the decade between 1977 and 1987 (1978–1979, 1982 and 1984–1986) with the highest peak in 1982 coincided with triphasic peaks of AP release [5,8,9].

In our four previous reports [5,6,7,8] published during 2003–2017, the author proposed that KD could be regarded as a pollen-induced disease (PID) of delayed-type hypersensitivity (DTH) with suppressed onset in the epidemics of influenza and subsequently conducted an epidemiological study on another vasculitis, Takayasu arteritis (TAK) or arteritis syndrome [9,10], which is a specific intractable disease (SID). First of all, the conventional bar graphs made from the registry of Japan Intractable Diseases Research Foundation showed only the changes in the number of presently RPs with TAK. Then, the author created line graphs showing the changes in the number of newly RPs with TAK during the period of 1975–2014, and demonstrated a previously unrecognized trend for an increase in TAK incidence in 1984.

Soon afterward, we began to draw merged line graphs showing the changes in the number of both presently and newly RPs for approximately 40 SIDs. Our first report [9] on SIDs presented epidemiological data on vasculitis syndrome (TAK, Behcet disease, thromboangiitis obliterans (Buerger’s disease), granulomatosis with polyangiitis (GPA) and periarteritis nodosa (PAN)), aplastic anemia and collagen diseases (systemic lupus erythematosus (SLE), rheumatoid vasculitis, scleroderma, sarcoidosis and pemphigus). Our second report [11] on SIDs showed epidemiological data on ulcerative colitis (UC), Crohn’s disease (CD), primary biliary cirrhosis (PBC), fulminant hepatitis with intractable hepatitis (FH), severe acute pancreatitis (SAP), interstitial pneumonia (IP), amyloidosis and idiopathic thrombocytopenic purpura (ITP). In these studies, monophasic outbreaks of SIDs in 1984–1986 were associated with increased AP levels. Furthermore, simultaneous outbreaks of those SIDs coincided with several subsequent peaks in pollen scatter from 1988 to 2014.

Our results [9,10,11] showed statistically significant correlations with Bonferroni correction between the annual number of newly RPs for all SIDs (*p* = 0.006) UC (*p* = 0.0003) and CD (*p* = 0.0008) in the patient-registry year and annual pollen levels measured 6 years prior to the patient-registry year.

Pollen cells consist of living sperm cells and pollen tube cells, having mt-DNA. The fact that the onset of different SIDs in Japan happened all together, being linked to airborne pollen exposure indicates that pollen might be one of the causative agents influencing on important pathways of the vital functions such as epigenetic alteration of metabolisms in various organs. Therefore, we speculated that cancer and malignant tumors might belong to another group of intractable diseases also triggered by pollen exposure. Thus, this is our first time to epidemiologically assess the cause of cancers using the same methodology as employed for our previous research [5,6,7,8,9,10,11] on the effect of pollen exposure for the occurrence of KD and various SIDs.

Visualization of only bar graphs or line graphs of the number of presently RPs with cancers might not predict environmental factors affecting disease onset or occurrence; however, plots of the annual change in newly RP after calculating the increase or decrease in the patient-registry numbers compared to previous years can help researchers to generate hypotheses regarding the causative factors of diseases. Furthermore, merged graphs for the number of both presently and newly RPs and AP exposure levels in three areas helped us to visualize these relationships. We have calculated the number of newly RPs each year from the annual number of presently RPs for all cancers and each of 23 types of cancers or malignant tumors, and have merged these graphs with pollen levels, to analyze their correlations over 20 years. This initial report describes the results for all cancers and five types of cancers; lung, stomach, colorectal, pancreatic and breast cancers.

## 2. Materials and Methods

Since 1958, the governmental authority in Japan, the NCC, has been gathering cancer incidence data by registering patients and releasing the data to the public [12].

The data shows the number of presently RPs in each present calendar year. In this study, we initially imported the number of presently RPs into the Excel tables. We then calculated the number of newly RPs in each year based on the annual number of presently RPs in some year and that in its previous year for all cancers and for each of 23 types of cancers or malignant tumors. The work was performed in accordance with the ethical principles for medical research outlined in the Declaration of Helsinki in 1964 and its subsequent revisions (https://www.wms.net/).

Data on AP release was provided by Dr. Yozo Saito, Dr. Hiroshi Yasueda and Professor Norio Sahashi [5]. Dr. Saito gathered AP data [5,8,9,10,11] based on the research unit in the Tokyo Medical and Dental University Graduate School of Medicine, Bunkyo-City, Tokyo, while Dr. Yasueda surveyed AP data [5,6,7,8,9,10,11] based on the research unit in the National Hospital Organization, Sagamihara National Hospital, Sagamihara, Kanagawa. As he ended his service of information transfer in 2006, no data has been obtained since then. The AP data in the Tokyo Metropolis collected from 12 research sites was donated by Mr. Hiroshi Kaneko. The AP data was downloaded after administrative information disclosures from the Tokyo Metropolitan Institute of Public Health website [13].

**Figure 1:**
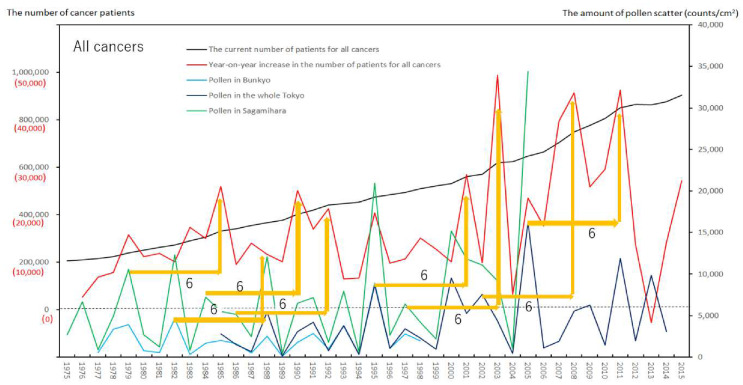
The current number of Japanese males and females (presently registered patients) registered for all cancers at the year diagnosed, its year-on-year increase (newly registered patients), and the amount of air borne pollen scatter in 3 areas during the period from 1975 to 2015. The line graphs for all cancers representing numbers of presently and newly registered patients in each year, as well as the amount of pollen scattered in Bunkyo-City area of Tokyo, the whole Tokyo Metropolitan area and Sagamihara City during the period from 1975 to 2015. Orange arrows indicate phasic increments of newly registered patients occurring six years after phasic increments of airborne pollen scatter. Numbers of patients are shown on the left axis whose scales consist of red numbers for newly registered patients and black numbers for presently registered patients. Pollen numbers in counts/cm^2^ are shown on the right axis.

**Figure 2:**
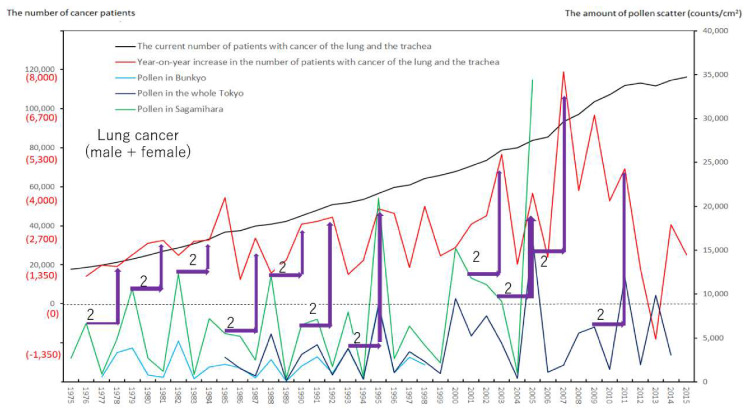
The current number of Japanese males and females (presently registered patients) registered for cancer of the lung and the trachea at the year diagnosed, its year-on-year increase (newly registered patients), and the amount of air borne pollen scatter in 3 areas during the period from 1975 to 2015. The line graphs for males and females with cancer of the lung and the trachea representing numbers of presently and newly registered patients in each year, as well as the amount of pollen scattered during the period from 1975 to 2015. Violet arrows indicate phasic increments of newly registered patients occurring two years after phasic increments of airborne pollen scatter.

**Figure 3:**
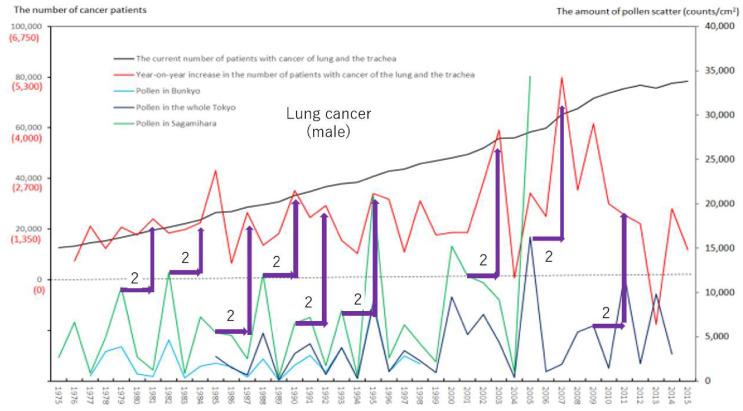
The current number of Japanese males (presently registered patients) registered for cancer of the lung and the trachea at the year diagnosed, its year-on-year increase (newly registered patients), and the amount of air borne pollen scatter in 3 areas during the period from 1975 to 2015. The line graphs for males with cancer of the lung and the trachea representing numbers of presently and newly registered patients in each year, as well as the amount of pollen scattered during the period from 1975 to 2015. Violet arrows indicate phasic increments of newly registered patients occurring two years after phasic increments of airborne pollen scatter.

**Figure 4:**
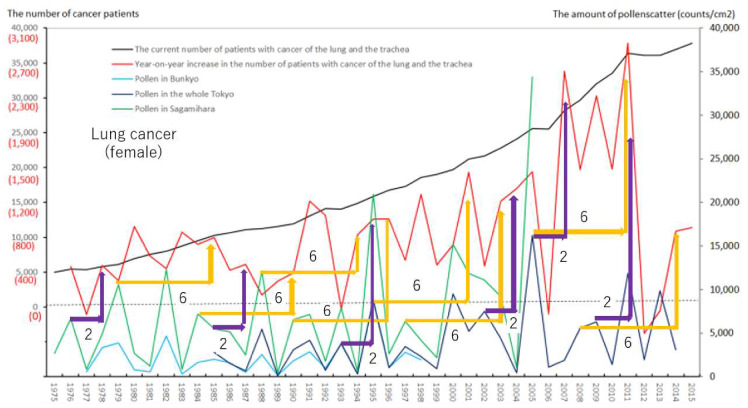
The current number of Japanese females (presently registered patients) registered for cancer of the lung and the trachea at the year diagnosed, its year-on-year increase (newly registered patients), and the amount of air borne pollen scatter in 3 areas during the period from 1975 to 2015. The line graphs for females with cancer of the lung and the trachea representing numbers of presently and newly registered patients in each year, as well as the amount of pollen scattered during the period from 1975 to 2015. Violet or orange arrows indicate phasic increments of newly registered patients occurring two or six years after phasic increments of airborne pollen scatter, respectively.

**Figure 5:**
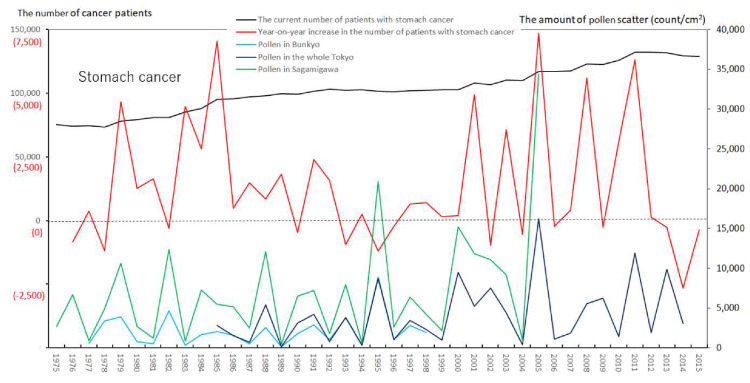
The current number of Japanese males and females (presently registered patients) registered for stomach cancer at the year diagnosed, its year-on-year increase (newly registered patients), and the amount of air borne pollen scatter in 3 areas during the period from 1975 to 2015. The line graphs for males and females with stomach cancer representing numbers of presently and newly registered patients in each year, as well as the amount of pollen scattered during the period from 1975 to 2015. No relationship was noted between phasic increments of newly registered patients and phasic increments of airborne pollen scatter.

**Figure 6:**
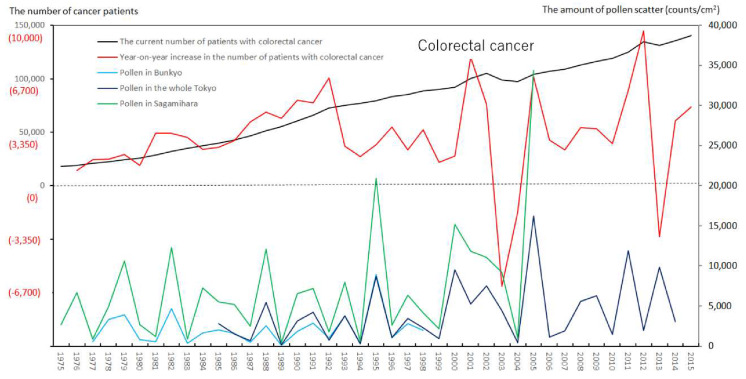
The current number of Japanese males and females (presently registered patients) registered for colorectal cancer at the year diagnosed, its year-on-year increase (newly registered patients), and the amount of air borne pollen scatter in 3 areas during the period from 1975 to 2015. The line graphs for males and females with colorectal cancer representing numbers of presently and newly registered patients in each year, as well as the amount of pollen scattered during the period from 1975 to 2015. No relationship was noted between phasic increments of newly registered patients and phasic increments of airborne pollen scatter.

**Figure 7:**
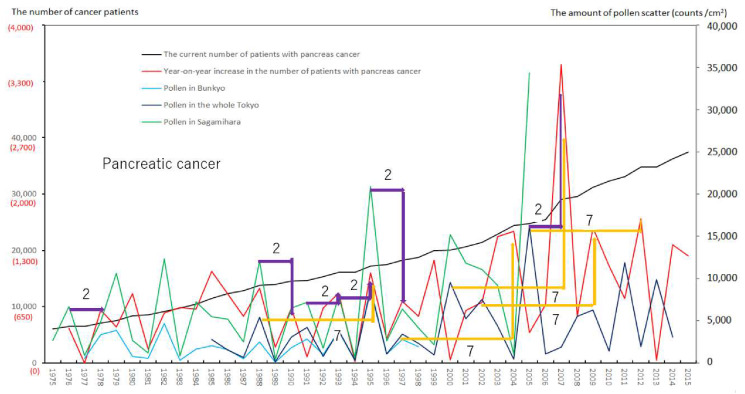
The current number of Japanese males and females (presently registered patients) registered for pancreatic cancer at the year diagnosed, its year-on-year increase (newly registered patients), and the amount of air borne pollen scatter in 3 areas during the period from 1975 to 2015. The line graphs for males and females with pancreas cancer representing numbers of presently and newly registered patients in each year, as well as the amount of pollen scattered during the period from 1975 to 2015. Violet or orange arrows indicate phasic increments of newly registered patients occurring two or seven years after phasic increments of airborne pollen scatter, respectively.

**Figure 8:**
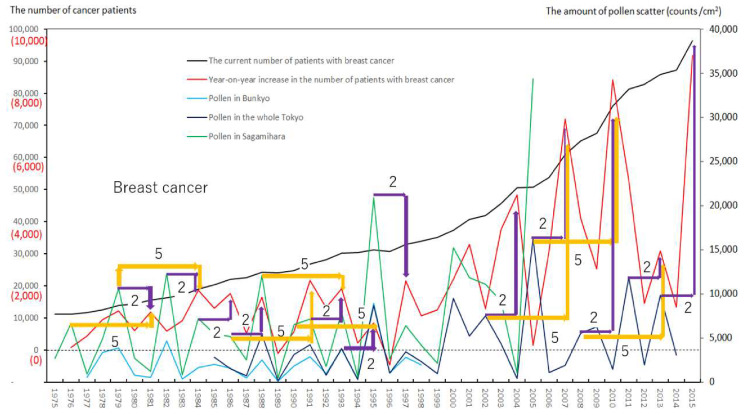
The current number of Japanese females (presently registered patients) registered for breast cancer at the year diagnosed, its year-on-year increase (newly registered patients) and the amount of air borne pollen scatter in 3 areas during the period from 1975 to 2015. The line graphs for females with breast cancer representing numbers of presently and newly registered patients in each year, as well as the amount of pollen scattered during the period from 1975 to 2015. Violet or orange arrows indicate phasic increments of newly registered patients occurring two or five years after phasic increments of airborne pollen scatter, respectively.

We created eight figures with line graphs indicating the annual number of presently and newly RPs for all cancers (Figure 1) and for each of the five cancers (Figure 2, Figure 3, Figure 4, Figure 5, Figure 6, Figure 7 and Figure 8). The five cancers included cancer of the lung and the trachea (lung cancer), as well as stomach, colorectal, pancreatic and breast cancers (Figure 2, Figure 3, Figure 4, Figure 5, Figure 6, Figure 7 and Figure 8). We also made the line graphs plotting the amount of AP scattered in three areas (Bunkyo-City area of Tokyo, the whole Tokyo Metropolitan area and Sagamihara City). With regard to lung cancer, three figures were created for male lung cancer (Figure 3), female lung cancer (Figure 4) and lung cancer in both males and females (Figure 2).

A correlation analysis was performed with Bonferroni correction for all cancers and for each of the five cancers between the annual number of newly RPs in each patient-registry year “x” (“x” = 1975–2015), and the annual AP levels in Tokyo and Sagamihara in the same year as “x” (“α” = 0). The correlation analysis was also performed with Bonferroni correction between the annual number of newly RPs in each patient-registry year “x” (“x” = 1975–2015), and the annual AP levels in both cities, measured “α” years prior to “x” (“α” = 1–7). Correlation coefficients and *p*-values were calculated with Bonferroni correction using the Excel function PEARSON as described at http://imnstir.blogspot.com/2014/04/p.htm. These shift analyses have been already done in our study of KD [7,10], and 40 SIDs [9,10,11], getting appropriate results. The *p* value for the correlations between cancer incidence and pollen levels was multiplied by 8, based on Bonferroni correction principle to avoid random correlations discovered. A statistically significant positive correlation was defined as *p* < 0.05 as shown in Table 1 (*p* < 0.001 ****, 0.001 ≤ *p* < 0.005 ***, 0.005 ≤ *p* < 0.01 ** and 0.01 ≤ *p* < 0.05 *). The correlation of positive tendency (0.05 ≤ *p* < 0.1 †) was also determined for reference.

## 3. Results

### 3.1. Upward Peaks in the Line Graphs of the Annual Number of Newly Registered Patients for All Cancers and for Each Cancer, in Relation to the Annual Levels of Airborne Pollen Scatter

Variations in the number of presently and newly RPs for all cancers and for each of lung, stomach, colorectal, pancreatic and breast cancers, are shown in Figure 1, Figure 2, Figure 3, Figure 4, Figure 5, Figure 6, Figure 7 and Figure 8. The total number of presently RPs for all cancers was 206,702 in 1975, 876,713 in 2014 and 903,914 in 2015 [12]. The total number of presently RPs for all SIDs was 17,595 in 1974, 21,694 in 1975, 925,646 in 2014 and 943,460 in 2015, respectively [9,10,11,14].

The five line graphs in our figures for all cancers (Figure 1) and for each of the five cancers (Figure 2, Figure 3, Figure 4, Figure 5, Figure 6, Figure 7 and Figure 8) included two line graphs demonstrating annual patient-registry data for both presently and newly RPs and three line graphs showing the annual AP scatter levels in the three areas. As shown in our line graphs in Figure 1, Figure 2, Figure 3, Figure 4, Figure 5, Figure 6, Figure 7 and Figure 8, the cedar pollen scatter in both Sagamihara City and Bunkyo-City started to increase in 1977–1987 in Japan, with three distinct peaks (1978–1980, 1982 and 1984–86) [5,8,9,10,11,15].

In association with the initial increase in Japanese AP levels beginning in 1978–1980, the number of newly RPs with all cancers showed the trend for increase, being similar to the number of newly RPs with KD, CD, and UD. As shown in Figure 1, the first trend for increase in newly diagnosed cases of all cancers occurred in 1978 (7822 cases), 1979 (15,795) and 1980 (11,206) [12] as likely as the increase in infant cases of KD [5,8,9,10,11,15,16] and in cases of all SIDs in 1978 (10,155), 1979 (10,287) and 1980 (5841) [9,10,11,14]. Subsequent larger increases in newly diagnosed cases of all cancers occurred in 1984 (15,046 cases) and 1985 (26,010) as likely as the increase in cases of all SIDs in 1984 (22,448) and 1985 (22,821) [9,10,11,14], while KD data showed a little different patterns characterized by the largest increase in 1982 and medium increase in 1984–1986 [5,8,9,10,11,15,16]. Figure 1 shows that the six phasic increments in the registry number of newly RPs for all cancers in 1978–1979, 1984–1985, 1989–1992, 2000–2003, 2006–2008 and 2010–2011.

In relation to the highest level of Japanese pollen release in 2005, shown in Tokyo and Sagamihara, as well as in other cities, the largest number of newly RPs during 1975–2015 was commonly shown for both all cancers and all SIDs in 2011, namely 6 years after 2005 [8,9,10,11,15,16]. The phasic increments of newly RPs were shown to occur after phasic increments of AP scatter; 6 years later in cases of all cancers (Figure 1), 2 years later in total cases of lung cancer (Figure 2), 2 years later in male cases of lung cancer (Figure 3), 2 and 6 years later in female cases of lung cancer (Figure 4), 2 and 7 years later in cases of pancreatic cancer (Figure 7) and 2 and 5 years later in cases of breast cancer (Figure 8), respectively.

### 3.2. Statistical Relationships between Newly Registered Patients in Each Patient-Registry Year and Airborne Pollen Levels Measured in the Same Year as or Prior to the Patient-Registry Year

We examined the statistical correlations for all cancers and for each of the five cancers between the annual number of newly RPs in each patient-registry year “x” (“x” = 1975–2015) and the annual AP levels in Tokyo and Sagamihara in the same year as “x” (α = 0) or “α” years prior to “x” (“α” = 1–7). Statistically significant positive correlations (*p* < 0.05) and reference positive tendencies (0.05 ≤ *p* < 0.1) are shown in Table 1. The blanks in Table 1 indicate non-significant results.

Our results showed no statistically significant correlation for any of cancers studied between the newly RPs in patient-registry year “x” and the AP exposure in the same year as “x.” In the shift analysis condition, significant correlations were shown between new occurrence of pancreatic cancer (*p* = 0.03) and breast cancer (*p* = 0.008) in the patient-registry year “x”, and AP levels in Tokyo measured 2 years prior to “x”, and between new occurrence of male and female lung cancer (*p* = 0.008), male lung cancer (*p* = 0.02), pancreatic cancer (*p* = 0.00003) and breast cancer (*p* = 0.002), and AP levels in Sagamihara measured 2 years prior to “x”. Furthermore, we found significant correlation between new occurrence of all cancers (*p* = 0.04) in the patient-registry year “x”, and AP levels in Sagamihara measured 6 years prior to “x”, and between new occurrence of breast cancer (*p* = 0.04) in patient-registry year “x”, and AP levels in Sagamihara measured 5 years prior to “x”. Additionally, positive tendencies were observed between new occurrence of all cancers (*p* = 0.08) in patient-registry year “x” and AP levels in Tokyo measured 6 years prior to “x”, between new occurrence of female lung cancer (*p* = 0.07) in “x” and AP levels in Sagamihara measured 2 and 6 years prior to “x”, and between new occurrence of pancreatic cancer (*p* = 0.05) in “x” and AP levels in Tokyo measured 7 years prior to “x”. Overall, all cancers, and each of lung, pancreatic, and breast cancers seemed to have a risk of new occurrence 2, and 5–7 years after the first sensitization and recognition of pollen exposure. Cumulative pollen exposure did not appear to contribute to the occurrence of stomach and colorectal cancers. Our analyses are summarized in Table 1.

## 4. Discussion

### 4.1. Trends in the Occurrence of Cancers Linked to Pollen Exposure

First of all, the Japanese history of forestry described below is important to understand our epidemiological data. Japanese cedar trees were rapidly cut on a large scale in the 1940-1950s, because of military and industrial needs during and after the World War II. Therefore, Japanese government strongly promoted a national forestation project in the 1950–1960s, and cedar trees were widely and rapidly planted in the entire Japanese land. In the 1970–1980s, namely 20 years after the period of cedar afforestation, the Japanese cedar trees gradually reached an age at which they have a high pollen producing capacity. Furthermore, cedar trees in Japan have been rarely cut since the 1990s, because of both increase in imported trees and decrease in demand for trees. Therefore, the Japanese cedar trees then have acquired even stronger pollen producing capacity. That is the background reason for the fact that Japanese pollen scatter began to increase during 1977–1987, characterized by three upward peaks (1978–1979, 1982 and 1984–1986) and continued to increase until 2005.

During the period of 1977–1987, when pollen release began to increase rapidly in Japan, KD in any of Tokyo, Kanagawa and all Japan showed triphasic outbreaks in 1978–1979, 1982 and 1984–1986, which coincided with triphasic peaks of AP scatter in Japan. Increases in all SIDs started in 1978–1979; many immune-mediated SIDs including TAK, Behcets disease, SLE, rheumatic vasculitis, scleroderma, pemphigus, UC, CD, IP and ITP showed the trend for increase commonly associated with the increasing AP release in 1984–1986. As shown in Figure 1, our study demonstrated an early trend for an increase in all cancers in 1978–1979 and in 1984–1985, as likely as the trend for increase in KD and all SIDs. A steady increase in the annual number of both presently and newly RPs with all cancers continued to occur from 1989, associated with increasing AP release up to 2015, as also observed for KD and multiple SIDs [5,8,9,10,11,15].

We hypothesized that a consecutive series of 13 AP peaks (1978–1979, 1982, 1984–1986, 1988, 1990–1991, 1993, 1995, 1997–1998, 2000–2002, 2005, 2008–2009, 2011 and 2013) might have produced the cumulative effects of epigenetic pollen exposure, which trigger immune-mediated diseases, when such cumulative effects overwhelmed the immune-reactive threshold for disease onset. To our knowledge, this is the first rapid communication of the association between pollen levels and the incidence of cancers. The number of newly RPs for all cancers in the patient-registry year “x” was correlated after Bonferroni correction with pollen levels measured 6 years prior to “x” (*p* = 0.04, Sagamihara), as was also observed for all SIDs (*p* = 0.006, Sagamihara), for UC (*p* = 0.0003, Sagamihara) and for CD (*p* = 0.008, Tokyo; *p* = 0.0008, Sagamihara) [11]. We found highly significant correlations between the number of newly RPs for lung, pancreatic and breast cancers in the patient-registry year “x” and pollen levels, measured 2 years prior to “x”.

Violet or orange arrows in Figure 1, Figure 2, Figure 3, Figure 4, Figure 7 and Figure 8 indicate that phasic increments of newly RPs come after phasic increments of AP scatter 6 years later for all cancers, 2 and 6 years later for lung cancer, 2 and 7 years later for pancreatic cancer and 2 and 5 years later for breast cancer. The statistical results summarized in Table 1 is totally compatible with these violet or orange arrows, which were drawn after painstaking work of tabulating pollen levels and cancer incidence over the four decades. The source for the annual number of presently and newly RPs was nationwide data in all Japan, while that for the annual AP levels was local surveillance data in Tokyo Metropolitan and in Sagamihara City. Statistical correlations between newly RPs and AP levels in Tokyo did not coincide wholly with those between newly RPs and AP levels in Sagamihara. Such inter-regional discrepancy in our statistical results is unlikely to be caused by different amount of AP levels between Tokyo and Sagamihara. Different period of AP measurements between Tokyo (during 1985–2014) and Sagamihara (during 1975–2005) might have affected our shift analysis study evaluating the effect of pollen exposure preceding the patient-registry on cancer development.

There are various kinds of very potent carcinogen, as listed by WHO. Although pollen cells are not believed to be usual carcinogens, our results suggest that pollen cells, living microparticles having mitochondria DNA may affect essential vital activities. The problems that remain to be solved are questions for antigen recognition mechanisms involved in triggering systemic reactions in various tissues and cells in the human body.

### 4.2. The Limits of Our Study, and Other Issues

The target of our present study was cancer that is multifactorial in causes and is a clinical condition defined at the cellular level by uncontrolled cellular mutation, invasive spread, metastases and escape from immune surveillance. Clinically, cancer can be diagnosed at various stages, ranging from the asymptomatic pre-cancerous stage, to the mildly or severely symptomatic stage, even at the postmortem stage. We therefore have to consider the effects of diagnostic advances such as in imaging tests, over the time frame described. CT scan started being used clinically in the early 1980s, MRI scans in 1990s and PET scans in 2000s. Screening programs including endoscopic tests to diagnose stomach/esophageal cancers were introduced in Japan over the time period of this study and could have influenced the number of cancers diagnosed. Advanced screening and imaging technology would lead to more accurate and effective diagnosis of cancers (increased detection of more early cancers with more pre invasive lesion).

Considering the lack of detail in the diagnosis (staging, tissue confirmed invasive cancers), we searched for the statistical correlation between newly diagnosed cancer patients and pollen exposure levels, rather than simply tracing total registration of cancer patients. The correlations between pollen levels and cancer incidence were drawn by use of the Bonferroni correction, to avoid running repeated correlations and getting some random correlations discovered. We multiplied the *p* value by 8, to keep the Bonferroni correction principle. As the limit of our study, we could not correct the data for possible confounders, such as age.

### 4.3. Considerations for Taking Measures against Environmental Risk of Pollen Exposure

Around 1980, we noted the presence of the FTS nonapeptide (zinc-free thymulin), which was first purified from blood as a serum thymic factor (FTS) by Bach et al. in 1977 [17]. We examined its effect using synthesized material in a lethal experimental allergic encephalomyelitis (EAE) model of guinea pigs for multiple sclerosis, a representative SID and demonstrated its dramatic preventive effect on lethality (Nagai Y, Abe H, Arita M. Medicine for demyelinating diseases. Tokkaisho 58-52225, Registered Patent No. JP.H0371413.B2, GB2109684.B.,1983) [18]. Based on these results, the author has further investigated the diverse immunological and pharmacological activities and biodefense effects of FTS nonapeptide (below, FTS nonapeptide), and found marked or considerable protective effects in many of animal models [19], such as aging in the senescence-accelerated mouse (SAM) model (JP.H01316328.A.) [20], lethality in X-ray radiated mice (Registered patent No 2655343.B2,EP.0377044.B1) [21,22], in D-variant of encephalomyocarditis (EMC-D) virus-induced diabetes and myocarditis in mice (JP.H01230529.A,EP0343258.B1) [23], in cardiomyopathic hamsters [24,25], in alloxan-treated diabetes in rats (JP.H012709839.B) [26], in drug-induced nephrotoxicity in mice (JP.H03178933.A.(Registered patent No.2655357.B2); JP.H06192120.A) [27,28,29], in drug-induced enteropathy in mice [30,31], in bleomycin-induced pulmonary fibrosis in mice [32], in adriamycin-induced cardiac toxicity in mice (JP.H06135849.A), in anti-tumor agent-induced white blood cell decreased model in mice (JP.H03204819.A, JP3159441.B2), in GVHD-induced model in mice (JP.3717955.B2), in virus-induced diseases in mice (JP.H01230529.A,EP0343258.B1;JP.H02306920) [33,34,35], in leukemia in mice [36] and in ovalbumin (OVA)-induced allergic rhinitis in guinea pigs [37], respectively. In allergic rhinitis as a pollinosis model [37], we observed a remarkable effect of FTS nonapeptide at a dose of 1/1000 that of prednisolone [37]. These findings demonstrated FTS nonapeptide as a biodefense substance of bio-origin with strong protective effects on various organ injuries, preferable redox-regulation effects, and non-toxic wide range of anti-inflammatory actions different from those of steroids, etc. (JP.5035582.B2(JP.WO2002017965.A1),EP.1316315.B1;JP.4721626.B2(2002.6.24.applied,2011.4.15.registered)). Pollen cells and their components have been recognized as the cause of various inflammation and allergies in allergic rhinitis/conjunctivitis (pollinosis), and we have noted environmental risks of AP exposure that potentially trigger the occurrence of many illnesses including KD, UC, CD and even cancers. Therefore, we have hopes for the FTS nonapeptide, which may be a potent intrinsic antagonist to protect against a wide range of inflammation and aging processes induced by pollen cells. Our interest has been the relationship between the FTS nonapeptide and sirtuin [38] in aging, as well as the effect of pollen exposure on many processes in which sirtuin is involved in the body. However, the gene(s) encoding the FTS nonapeptide and its related substances have not yet been identified.

The classic theory for cancer development is multi-stage carcinogenesis [39]. Extensive research by biologists and physicians specializing in each cancer using rodent models exposed to or immunized with pollens is needed to determine the mechanisms of these associations and to answer the question whether pollen substances affect precancerous stage or each of the three stages of carcinogenesis based on the multi-stage theory. A large cohort study is needed in the future to analyze the relationship between the onset of various cancers, SIDs and KD and AP exposure. Moreover, it is important to establish useful diagnostic parameters to track disease progression in addition to lymphocyte stimulation tests [40,41] with pollen substances in patients with cancers and SIDs.

The avoidance of pollen exposure shall be recommended to prevent the onset of cancers when people reach the susceptible age for cancer or when even younger patients have a family history or predisposition to cancer. People should be advised to take precautions to wear safety masks and goggles and to equip the room air cleaners, particularly during the spring season with large amounts of pollen scatter and even during the autumn season with a small amount of fore-running release of cedar pollen before the next spring. Furthermore, epidemiological studies are needed to determine whether pollen avoidance suppresses the occurrence of cancer progression and recurrence in patients diagnosed as cancer, during and after therapy. Recent advances in issues of artificial intelligence and informatic science related to public health management are expected to greatly contribute to improve current strategies of pollen avoidance. The final aim of our study was to determine the risk of AP exposure and its influence on the occurrence of immune-related diseases such as KD, UC, CD, PBC, IP, connective tissue diseases, vasculitis syndrome and cancers in Japan. Further studies are needed to make sure that these immune-related diseases and cancers might also be PIDs or “pollen diseases” whose conditions are triggered by epigenetic immune-mediated stress induced by pollen cells.

## Figures and Tables

**Table 1:** Statistical correlations between newly registered patients with cancer and airborne pollen levels.

α		All Cancers	Lung Cancer Male + Female)	Lung Cancer (Male)	Lung Cancer (Female)	Stomach Cancer	Colorectal Cancer	Pancreatic Cancer	Breast Cancer
0	T								
	S								
1	T								
	S								
2	T							0.03 *	0.008 **
	S		0.008 **	0.02 *	0.07 †			0.00003 ****	0.002 ***
3	T								
	S								
4	T								
	S								
5	T								
	S								0.04 *
6	T	0.08 †							
	S	0.04 *			0.07 †				
7	T							0.05 †	
	S								

Correlation analysis with Bonferroni correction between the annual number of newly registered patients in each patient-registry year “x” (“x” = 1975–2015), and the annual airborne pollen levels in Tokyo (T) and Sagamihara (S) in the same year as the patient-registry year “x” (α = 0) and “α” years, prior to the patient-registry year “x” (α = 1–7) for all cancers, male and female lung cancer, male lung cancer, female lung cancer, stomach cancer, colorectal cancer, pancreatic cancer and breast cancer. Statistically significant positive correlations (*p* < 0.001 ****, 0.001 ≤ *p* < 0.005 ***, 0.005 ≤ *p* < 0.01 ** and 0.01 ≤ *p* < 0.05 *), and reference positive tendency results (0.05 ≤ *p* < 0.1 †) are shown. Blanks indicate non-significant correlations.

## Abbreviations

AP	airborne pollen
CD	Crohn’s disease
DTH	delayed-type hypersensitivity
FH	fulminant hepatitis with intractable hepatitis
GPA	granulomatosis with polyangiitis
IBD	inflammatory bowel disease
IP	interstitial pneumonia
ITP	idiopathic thrombocytopenic purpura
KD	Kawasaki disease
LST	lymphocyte stimulation test
NCC	National Cancer Center
PAN	periarteritis nodosa
PBC	primary biliary cirrhosis
PE	pollen exposure
PID	pollen-induced diseases
RPs	registered patients
Sagamihara	Sagamihara City in Kanagawa Prefecture
SAP	severe acute pancreatitis
SIDs	specific intractable diseases
SLE	systemic lupus erythematosus
TAK	Takayasu arteritis
UC	ulcerative colitis7
